# Influence of water content in mixed solvent on surface morphology, wettability, and photoconductivity of ZnO thin films

**DOI:** 10.1186/1556-276X-9-485

**Published:** 2014-09-11

**Authors:** Min Zhao, Fengjiao Shang, Jianguo Lv, Ying Song, Feng Wang, Zhitao Zhou, Gang He, Miao Zhang, Xueping Song, Zhaoqi Sun, Yiyong Wei, Xiaoshuang Chen

**Affiliations:** 1 School of Physics and Material Science, Anhui University, Hefei 230039, China; 2 School of Electronic and Information Engineering, Hefei Normal University, Hefei 230601, China; 3 National Laboratory for Infrared Physics, Shanghai Institute of Technical Physics, Chinese Academy of Sciences, Shanghai 200083, China

**Keywords:** Thin films, Chemical synthesis, Electron microscopy, Electrical conductivity

## Abstract

ZnO thin films have been synthesized by means of a simple hydrothermal method with different solvents. The effect of deionized water content in the mixed solvents on the surface morphology, crystal structure, and optical property has been investigated by scanning electron microscopy, X-ray diffraction, and UV-Vis spectrophotometer. A large number of compact and well-aligned hexagonal ZnO nanorods and the maximal texture coefficient have been observed in the thin film, which is grown in the mixed solvent with *x* = 40%. A lot of sparse, diagonal, and pointed nanorods can be seen in the ZnO thin film, which is grown in the 40-mL DI water solution. The optical band gap decreases firstly and then increases with the increase of *x*. Reversible wettability of ZnO thin films were studied by home-made water contact angle apparatus. Reversible transition between hydrophobicity and hydrophilicity may be attributed to the change of surface chemical composition, surface roughness and the proportion of nonpolar planes on the surface of ZnO thin films. Photocurrent response of ZnO thin films grown at different solvents were measured in air. The response duration of the thin film, which is grown in the solvent with *x* = 40%, exhibits a fast growth in the beginning but cannot approach the saturate current value within 100 s. The theoretical mechanism for the slower growth or decay duration of the photocurrent has been discussed in detail.

## Background

ZnO have received increasing attention due to its wide band gap (3.37 eV) and large excitonic binding energy (60 meV), which is promising for fabricating nanoscale electronic, optical, optoelectronic, electrochemical, and electromechanical devices [1,2]. Recently, surface wettability and photocurrent property of low-dimensional ZnO nanostructures have been studied by some research group [3-5]. A variety of methods have been employed to fabricate ZnO thin films, such as molecular beam epitaxy (MBE) [6], chemical vapor deposition (CVD) [7], sol-gel process [8], spray pyrolysis [9], magnetron sputtering [10], and hydrothermal synthesis [11,12]. Among all these methods, the hydrothermal synthesis may be the most simple, low-cost, and effective way to control the surface morphology of ZnO at relatively low temperatures, while exempted from further annealing. For the hydrothermal synthesis, the solvents have a considerable influence on the final nanostructure size and morphology of the as-prepared ZnO thin films. So far, most of the studies on ZnO prepared by this method mainly employed single liquid (such as water, methanol, ethanol, and acetone) as solvent. However, studies on the surface morphology and photoelectric properties of ZnO by using two kind of liquid as a mixed solvent are still inadequate. Therefore, it is necessary to study the relationship between the ratio of solvents and surface morphology and properties of ZnO.

In this letter, ZnO thin films were synthesized using the mixed solvents of deionized (DI) water and ethylene glycol monomethyl ether by hydrothermal method. The effects of the DI water content in the mixed solvents on surface morphology, optical property, wettability, and photocurrent property were explored.

## Methods

All chemicals were of analytical reagent grade and used as received without further purification. Polished silicon wafers (15 × 15 mm^2^), which were cleaned successively in acetone and DI water for 10 min in anultrasonic bath, were used as substrates. ZnO seed layer was prepared by sol-gel process. Ethylene glycol monomethyl ether [C_3_H_8_O_2_] and monoethanol amine (MEA) were used as the solvent and stabilizing agent, respectively. Zinc acetate dihydrate [Zn(CH_3_COO)_2_ 2H_2_O] was dissolved in a mixture of ethylene glycol monomethyl ether and MEA at room temperature. The obtained solution was stirred using magnetic stirrer at 60°C for 2 h to obtain clear and homogeneous solution. The ZnO seed layer was prepared by spin-coating technology with a rate of 3,000 rpm for 30 s. After each spin coating, the seed layer was kept at 150°C for 10 min and this procedure was repeated two times. The seed layer was annealed at 600°C for 1 h and then cooled down to RT. Subsequently, ZnO thin films were synthesized by hydrothermal method on the ZnO seed layer. Zinc nitrate hexahydrate (Zn(NO_3_)_2_ · 6H_2_O) and equimolar hexamethylenetetramine (C_6_H_12_N_4_, HMT) dissolved in 40 mL-mixed solvents of DI water and ethylene glycol monomethyl ether (EGME). The concentration of zinc nitrate hexahydrate was 0.06 mol/L. The DI water content *x* in the mixed solvents was 0%, 40%, 80%, and 100%, respectively. The solution was transferred to a 50-mL Teflon-lined stainless steel autoclave (Machinery Factory of USTC, Hefei, China) and the ZnO seed layer was placed in the bottom of the autoclave. Growth of ZnO thin film was carried out at 115°C for 2 h in an oven. After growth, the as-prepared ZnO thin film was removed from the solution, rinsed with DI water, and dried at 60°C.

Surface morphology of the ZnO thin films were observed by scanning electron microscopy (SEM, SU1510). Microstructure of the ZnO thin films were measured by X-ray diffraction (XRD, MACM18XHF) with Cu*Kα* radiation (λ =0.15405 nm). Absorption spectra of the thin films were measured by UV-Vis spectrophotometer. Water contact angles (WCAs) were investigated by home-made WCA apparatus at room temperature. DI water droplet with a volume of 5 μL was used in the wettability test. WCAs were measured at four different positions of a sample, and the average values of the WCAs were reported. A 36-W UV lamp, centered at 254 nm, was used to study the effect of UV irradiation on the wettability of the samples. Light-induced hydrophilicity was evaluated in atmospheric air by irradiating the samples at certain time intervals. After each irradiation time interval, a 5-μL DI water droplet was placed on the irradiated area and the corresponding contact angle was measured. The reverse transition from the hydrophilic to hydrophobic states could be performed via the storage in dark conditions at room temperature. The photocurrent response of the ZnO thin films to UV light was measured at 5-V bias voltages by switching the 365-nm UV light source with an intensity of 1 μW/cm^2^ using a CHI 650 electrochemical workstation (CH Instruments, Chenhua Co., Shanghai, China) at room temperature in ambient conditions.

## Results and discussion

**Figure 1:**
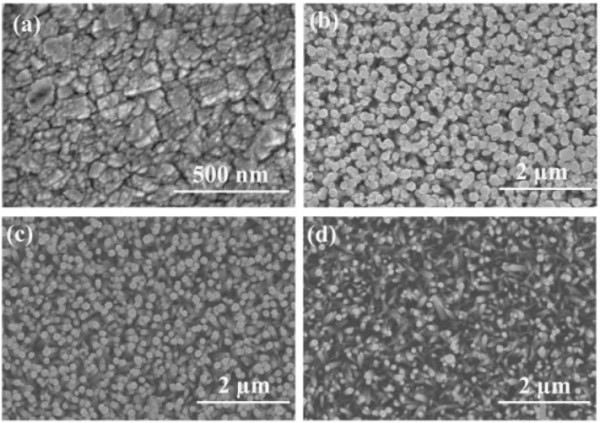
**Typical SEM images of ZnO thin films (a) *x*** **= 0%, (b) *x*** **= 40%, (c) *x*** **= 80%, (d) *x*** **= 100%.**

**Figure 2:**
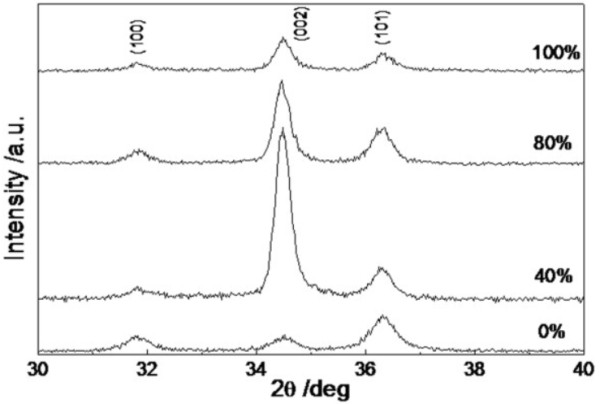
XRD patterns of ZnO thin films.

SEM images of ZnO thin films, which have been prepared in different solvents, are shown in Figure 1. When the thin film grows in the solvent of 40 mL ethylene glycol monomethyl ether, a lot of compact nanoparticles have been observed on surface of the thin films. A large number of compact and well-aligned hexagonal ZnO nanorods have been observed on the surface of the thin film, which grows in the mixed solvent with *x* = 40%. ZnO nanorods have an average diameter of approximate 100 nm. The result indicates that the ZnO thin film is highly oriented along the *c*-axis. As DI water content further increases, average diameter, density, and order degree of the ZnO nanorods decrease. Figure 2 shows surface morphology of the ZnO thin film grows in the 40-mL DI water solution. It can be seen that the thin film is not well ordered and composed of a lot of sparse, diagonal, and pointed nanorods. Different surface morphologies of the thin films can be attributed to different nucleation and growth mechanisms. The nucleation and growth habit of polar ZnO crystal under hydrothermal condition has been investigated by many researchers. It has been found that the polarity and saturated vapor pressure of the solvents, precursor, reaction temperature and time, and solution basicity have significant effects on the surface morphology of ZnO [11,13,14]. It is well known that the growth rate along polar (0001) crystal plane of hexagonal wurtzite ZnO crystal is relatively faster than that of nonpolar crystal plane. Therefore, the growth rate along the *c*-axis direction is much faster than other crystallographic directions. In our case, the ZnO thin films are grown in a mixed solvent with different DI water contents at 115°C. The boiling point of DI water and EGME is 100°C and 125°C, respectively. Therefore, diversity of surface morphology of the ZnO thin films may be attributed to the difference of the polarity and saturated vapor pressure of the solvent. When *x* =0, the ZnO thin film is grown in the EGME solution with a lower saturated vapor pressure and lesser polarity, which is hard to form an individual pattern such as rodlike ZnO [13]. Therefore, the ZnO thin film is composed of irregular nanoparticles. The ZnO thin films are grown in the mixed solvent of EGME, and DI water consists of well-aligned hexagonal ZnO nanorods, which may be due to the greater polarity and higher saturated vapor pressure of the solvents. The dispersion of the precursor in water was not better than in organic solvents. The morphology of ZnO thin film synthesized in water was less homogeneous than that prepared in organic solvents [14]. That is the reason why the ZnO thin film grown in DI water consists of sparse, diagonal and pointed nanorods.

**Figure 3:**
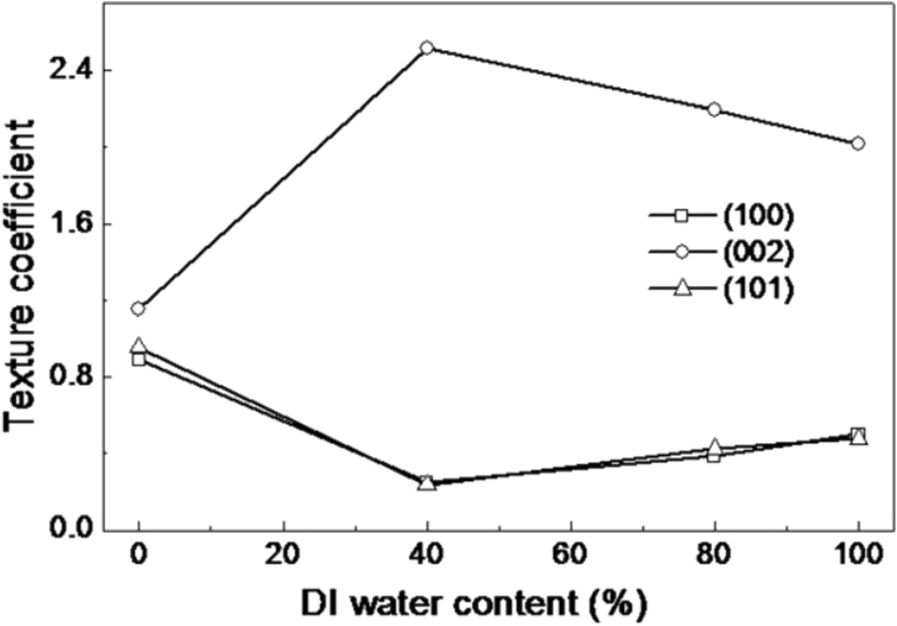
Texture coefficient of ZnO thin films versus water content.

Figure 2 shows the XRD patterns of ZnO thin films. It can be seen that the three diffraction peaks at 2*θ* = 31.77°, 34.42°, and 36.25° in the XRD patterns are indexed to (100), (002), and (101) hexagonal wurtzite structure ZnO (JCPDS 36-1451), respectively. When *x* = 0%, no preferred orientation has been observed in the thin film. As *x* increases from 0% to 100%, the relative intensity of (002) diffraction peak increases first and then decreases, indicating that preferred orientation along *c*-axis of the ZnO thin films enhances and then reduces. The texture coefficient (TC), which can be used to characterize quantitatively the preferred orientation of the thin film, has been calculated by using the following formula [15]:

(1) $${\mathrm{TC}}_{\left(\mathrm{hkl}\right)}=\frac{I_{m}\left(\mathrm{hkl}\right)/I_{0}\left(\mathrm{hkl}\right)}{\left(1/n\right)\sum I_{m}\left(\mathrm{hkl}\right)/I_{0}\left(\mathrm{hkl}\right)}$$

where *I*_m_(*hkl*) is the measured relative intensity of the reflection from the (*hkl*) plane, *I*_0_(*hkl*) is that from the same plane in standard reference data (JCPDS 36-1451), *n* is the number of reflection peaks from the film. In our case, *n* = 3 because three major reflection peaks come from (100), (002), and (101) plane and are involved. For the extremely preferential orientation, *T*(*hkl*) = 3, while for the random one, *T*(*hkl*) = 1. The texture coefficients, TC(100), TC(002), and TC(101), of ZnO thin films versus water content are shown in Figure 3. It can be seen that the three texture coefficients of the thin film, which grow in the mixed solvents with *x* = 0%, is approximately equal to 1. This tells us that the thin film is randomly oriented. Increasing the DI water content, TC(002) of the thin films increases first, reaches a maximum at *x* = 40%, and then decreases. The results indicate that the thin films grown in the mixed solvents with *x* = 40% exhibit the best *c*-axis-preferred orientation.

**Figure 4:**
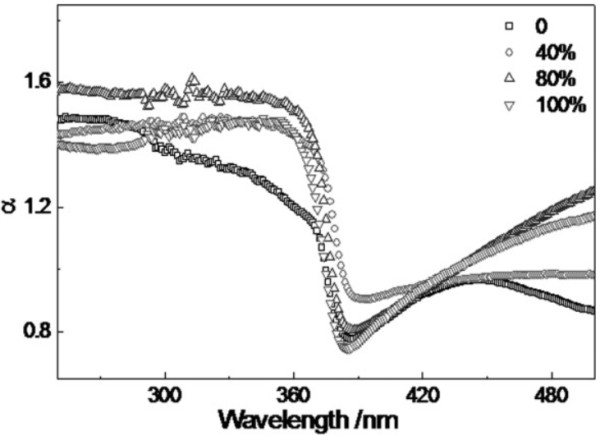
Absorption spectra of ZnO thin films with different DI water content.

**Figure 5:**
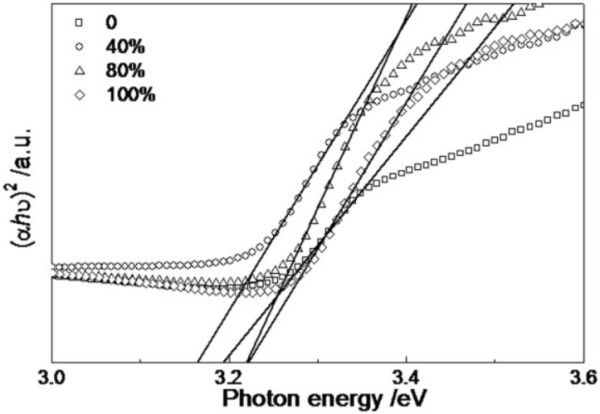
**Plots of (*αhν *)**^**2 **^**versus *hν *** **of ZnO thin films.**

Absorption spectra of the ZnO thin films with different DI water content are shown in Figure 4. It can be seen that all of the absorption spectra exhibit an abrupt absorption edge at about 380 nm, and absorption edge of the thin films shifts to long-wavelength region (red-shift) first and then shifts to short-wavelength region (blue-shift) with the increase of *x*. As a direct band gap semiconductor, the relationship between absorption coefficient (*α*) of the thin films and phonon energy (*hν*) can be obtained by applying the following formula [16]:

(2) $${\left(\mathrm{\alpha h\nu}\right)}^{2}=A\left(\mathrm{h\nu}-E_{g}\right)$$

where *E*_*g*_ is the optical band gap of the thin films and A is a constant. Figure 5 shows the plots of (*αhν*)^2^ versus *hν* of the thin films. The optical band gap can be calculated by extrapolating the straight linear portion of the plots between (*αhν*)^2^ and *hν* to the phonon energy axis [16]. The optical band gaps of the thin films are shown in Table 1. It can be seen that the value of optical band gap decreases first and then increases with the increase of *x*. Rao et al [17] thought that the compressed lattice is expected to provide a wider optical band gap because of the increased repulsion between the oxygen 2p and the zinc 4 s bands. The decrease of the optical band gap of the ZnO thin films could be attributed to the decrease of compressive stress in the thin films. The reduced compressive stress in the thin film may be attributed to suppression of grain boundary by the formation of ZnO nanorods. Because the grain boundaries are planar defects and have a strained bonding status, the elimination of grain boundaries is expected to mitigate the strain remaining in the films [18]. In our case, the minimal optical band gap, which may be due to the well-aligned hexagonal ZnO nanorods, has been observed in the ZnO thin film grown in the mixed solvents with *x* = 40%.

**Table 1:** Optical band gap and contact angle reduction rate of ZnO thin films

***x *** **(%)**	***E***_***g***_	**Contact angle reduction rate**
0	3.18	0.847
40	3.17	0.789
80	3.21	0.891
100	3.26	0.922

**Figure 6:**
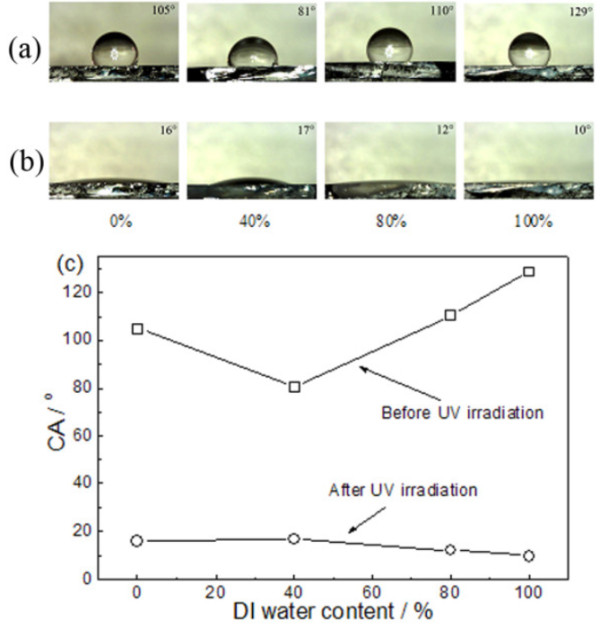
**Images of water droplet shape on the ZnO thin films before (a) and after (b) UV irradiation for 120 min.** The corresponding WCA was plotted in **(c)**.

Figure 6a shows the typical droplet shape of a 5-μL droplet on the ZnO thin films. It can be seen that the WCA of the thin films, which are grown in mixed solvent with DI water content *x* of 0%, 40%, 80%, and 100%, are 105°, 81°, 110°, and 129°, respectively. It is well known that the wettability of a solid surface can be governed by both the surface chemical composition and the geometrical structure of the surfaces. On a rough solid surface, air can be trapped in the space of solid surface and form a composite interface of water/solid and water/air between solid and droplet. The hydrophobicity of a rough surface can be enhanced by increasing the proportion of water/air interface. ZnO has a hexagonal wurtzite crystal structure in which each Zn^2+^ ion is surrounded by a tetrahedron of four O^2-^ ions and vice versa. This structure can be described schematically as a number of alternating planes of O and Zn ions stacked along the *c*-axis [19]. The nonpolar planes are formed by breaking the same number of oxygen and zinc bonds, and they contain an equal number of O and Zn ions, whereas the polar planes are either Zn- or O-terminated [20]. Surface free energy of ZnO thin films are proportional to the ratio of polar planes on the surface. It can be seen from SEM images that as the *x* increases from 0% to 100%, spaces between ZnO nanoparticles on the surface gradually increase and polar planes and corresponding surface free energy of the thin films increase first and then decrease. Therefore, the variety of WCA could be mainly attributed to the change of surface morphology of the ZnO thin films. Figure 6b shows the typical droplet shape of a 5-μL droplet on the ZnO thin films after an exposure to UV irradiation for 120 min. The results indicate that all the samples exhibit obvious photo-induced transition in their wettability to hydrophilicity, and the WCA increases first and then decreases with the increase of *x*. In order to evaluate the efficiency of photo-induced process [21], the contact angle reduction rates are calculated by using the following relation:

(3) $$\begin{array}{l} \mathrm{Contact}\;\mathrm{angle}\;\mathrm{reduction}\;\mathrm{rate}\\ \;=\left({\left(\mathrm{WCA}\right)}_{t=0}-{\left(\mathrm{WCA}\right)}_{t=120}\right)/{\left(\mathrm{WCA}\right)}_{t=0} \end{array}$$

(WCA)_*t* = 0_ and (WCA)_*t* = 120_ are the WCA value of ZnO thin films before and after UV irradiation for 120 min. The results are listed in Table 1. It can be seen that the largest photo-induced transition appear in the ZnO thin film with *x* = 100%. It should be emphasized that the changes of the wettability of the thin films are reversible by storage in dark [21].

It is known that UV irradiation can generate electron-hole pairs in the ZnO lattice. These electrons and holes can either recombine or move to the surface to react with species adsorbed on the surface [22]. Some of the holes react with lattice oxygen to form surface oxygen vacancies, while the electrons can react with the lattice zinc ions (Zn^2+^) to form surface Zn^+^ defective sites [23]. Water and oxygen may compete to dissociatively adsorb on these defective sites. The defective sites are kinetically more favorable for hydroxyl adsorption than oxygen adsorption. As a result, the surface hydrophilicity is improved [24]. It has also been presented that the surface becomes energetically unstable after the hydroxyl adsorption. The oxygen adsorption is thermodynamically favored; thus, oxygen can form stronger bonds to the defective sites than the hydroxyl group. Consequently, the hydroxyl groups adsorbed on the defective sites can be replaced gradually by oxygen atoms when the samples are placed in the dark [21]. Therefore, the surface evolves back to its original state before UV irradiation, and the wetting property is reconverted from hydrophilicity to hydrophobicity.

Wettability transition of the samples suggest that the wetting model switches from the Cassie-Baxter state to the Wenzel state [21], as the latter is the model predicting the possibility of superhydrophilicity of the thin film with very rough surfaces. This indicates that the surface chemical composition and surface roughness play an important role in the photo-induced process. The former provides a photosensitive surface, which can be used to control the wetting states, while the latter further enhances the wettability. In addition, the photo-induced hydrophilicity may be related to the proportion of nonpolar planes on the surface. On the nonpolar planes, both oxygen and zinc ions are terminated in the same plane. These surface oxygen ions are energetically more reactive than their surrounding atoms, and considered to act as reactive sites for increasing OH species on the surface. Photo-induced contact angle reduction rate of nonpolar plane-oriented ZnO thin films was faster than that of polar plane-oriented ZnO thin films [25]. That is the reason of ZnO thin film grown in 40-mL DI water solution has the largest contact angle reduction rate, while the ZnO thin film grown in the solvent with *x* = 40% has the minimal contact angle reduction rate. Therefore, the reversible transition between hydrophobic and hydrophilic properties can be controlled by the surface chemical composition, the surface roughness, and the proportion of nonpolar planes on the surface.

**Figure 7:**
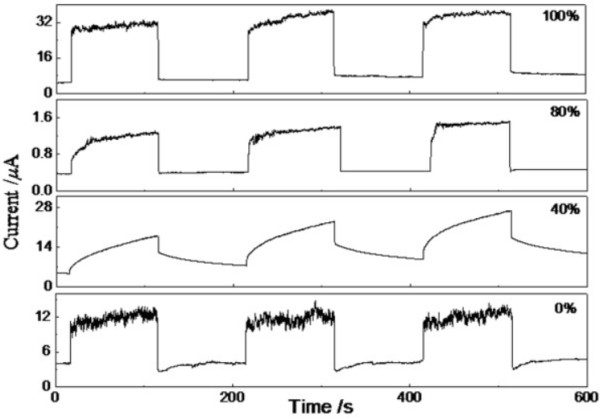
Photocurrent growth and decay under the periodic (100 s) illumination of UV light at 5-V bias voltages.

Photocurrent properties of ZnO thin films grown at different solvents with and without UV light irradiation were measured in air between two Ag electrodes under a bias voltage of 5 V. The time-dependent photocurrent curve of ZnO thin films with UV light turned on-off are shown in Figure 7. It can be seen that the three thin films grown in the solvents with *x* = 0%, 80%, and 100% have a fast growth and decay when the UV light is turned on-off. The fast growth and decay of photocurrent is mainly attributed to the adsorption and desorption of oxygen molecules on the surface of ZnO thin films. In the dark, oxygen molecules are adsorbed on the surface and capture free electrons from the thin films, creating surface oxygen ions (O^2-^) and forming a depletion layer near the surface and thus reducing the conductivity [26]. When the thin films are exposed to UV light with photon energies above the band gap of ZnO, electron-hole pairs are generated by the absorption of a photon. The holes are then driven by the depletion field to the surface and combine with the surface oxygen ions (O^2-^). The increased free carrier concentration and the decreased depletion width near the surface result in an enhancement of the conductivity [27]. The better photoresponse of the thin film with *x* = 100% could be attributed to the enhanced light absorption and surface-state trapping due to the larger surface-to-volume ratio [27,28]. The response duration of the thin film grown in the solvent with *x* = 40% following a fast growth in the beginning but cannot approach the saturate current value within 100 s. The photocurrent decays following a fast decline in the beginning and has a long tail close to the dark current. In this case, the exchange process for carrier trapping at the surface states due to oxygen absorption or desorption on the surface of ZnO thin film makes one part of the growth or decay duration. Apart from the oxygen absorption or desorption, there is another exchange process for carrier recombination at the deep defect level to make the other part of the growth or decay duration [29]. For ZnO thin film, there also exists interactions between the oxygen gas and the native defect states, which are much slower than the surface process due to higher activation energy. Thus, slower growth or decay duration of the photocurrent occurs and then forms the corresponding slower component of the transient process [27].

## Conclusions

In the present work, surface morphology, microstructure, optical absorption spectra, and WCA of ZnO thin films, which are grown in solutions with different DI water contents as solvents, were measured by SEM, XRD, UV-Vis spectrophotometer, and home-made WCA apparatus. The results indicate that the surface morphology, *c*-axis-preferred orientation, and optical band gap of the thin films are closely related to the DI water content. The maximal WCA before UV irradiation and the largest WCA reduction rate after 120-min UV irradiation have been observed in the thin film with *x* = 100%. The response duration of the thin film grown in the solvent with *x* = 40% attributed to oxygen absorption or desorption on the surface and interactions between the oxygen gas and the native defect states of ZnO thin film.

## Competing interests

The authors declare that they have no competing interests.

## Authors’ contributions

This idea is from JL, ZS, and XC. MZ, FS, YS, FW, and ZZ fabricated the ZnO thin films. MZ, FS, YW, FW, ZZ, and MZ measured all the thin films under the instruction of GH and XS. All authors contributed to the revision of the manuscript. All authors read and approved the final manuscript.
